# Supplementary material for: Cloud BioLinux: pre-configured and on-demand bioinformatics computing for the genomics community
Source: BMC Bioinformatics. 2012 Mar 19;13:42. doi: 10.1186/1471-2105-13-42 (PMC3372431; doi:10.1186/1471-2105-13-42)
Supplement: Additional file 1 — Supplementary 1 Cloud BioLinux software documentation in the form of a mini, self-contained website. Users need to download and uncompress the .zip file, and open through a web browser the "index.html" file available on the main directory. (ZIP 1823 kb). [file 1471-2105-13-42-S1.ZIP › Cloud-BioLinux-Package-Documentation/docs/sibsim4.html]

Bio-Linux Software Documentation Pages

Back to search form

## sibsim4

|  |  |
| --- | --- |
| Name | sibsim4 |
| Description | The sibsim4 project is based on sim4, which is a program designed to align an expressed DNA sequence with a genomic sequence, allowing for introns. sibsim4 provides an extensive revision of the original code the following goals:  - speed improvement;- allow large, chromosome scale, DNA sequences to be used;- provide more detailed output about splice types;- provide more detailed output about polyA sites;- misc code cleanups and fixes.  The sibsim4 package is prepared by Debian-Med and can be installed in Bio-Linux by typing `sudo apt-get install sibsim4` For further information please see the remote documentation links |
| Homepage | http://sibsim4.sourceforge.net/ |
| Remote Documentation | http://sibsim4.sourceforge.net/manpage.html |
